# Accuracy of Digital Impressions of Implants With Angulated and Different Vertical Positions in the Full Arch: An In Vitro Study

**DOI:** 10.1155/ijod/9747675

**Published:** 2026-06-30

**Authors:** Sina Safari, Neshat Atashpareh, Hamed Bahrami Maleki

**Affiliations:** ^1^ Department of Prosthodontics School of Dentistry, Kerman University of Medical Sciences, Kerman, Iran, kmu.ac.ir; ^2^ Department of Prosthodontics School of Dentistry, Shiraz University of Medical Sciences, Shiraz, Iran, sums.ac.ir; ^3^ Department of Prosthodontics School of Dentistry, Ilam University of Medical Sciences, Ilam, Iran, medilam.ac.ir

**Keywords:** digital impression, full-arch implant, intraoral scanner, precision, trueness

## Abstract

**Purpose:**

The present in vitro study was conducted to evaluate the trueness and precision of full‐arch digital impressions of implants with different angulations and vertical positions.

**Methods:**

Two angulated and two parallel fixtures were placed on two typodonts (subcrestal and crestal). Four scan bodies were fixed on the fixtures. Typodonts were scanned using 3Shape TRIOS 3 and Planmeca Emerald scanners. Furthermore, they were also scanned by a 3Shape D810 laboratory scanner as a reference. Subsequent to making a cross‐section on the scan bodies, four points were obtained, out of which, six lines were traced. The obtained lines represented the distances between scan bodies. The differences of the distances in the scans prepared by the TRIOS 3 and Planmeca Emerald scanners were measured using the reference scanner and were analyzed using a paired *t*‐test.

**Results:**

In subcrestal models, TRIOS 3 showed significant differences for distances 1–2, 1–3, and 2–4, while Planmeca Emerald showed significance only for 2–4. In crestal models, TRIOS 3 showed significant differences for 1–3, 1–4, 2–4, and 3–4, whereas Planmeca Emerald showed significance only for 2–4 after correction.

**Conclusions:**

The TRIOS 3 scanner showed higher trueness and precision in subcrestal and angulated implants, while the Planmeca Emerald showed slightly higher trueness at the crestal implants. Both scanners showed small deviations relative to the reference scanner, with TRIOS 3 demonstrating higher overall precision.

## 1. Introduction

Recent advances in digital dentistry have transformed clinical workflows, enabling clinicians to provide more predictable and high‐quality care. Dental implants are widely used to rehabilitate partially or fully edentulous patients, and implant‐supported fixed prostheses have been shown to improve the function, esthetics, and overall quality of life. Successful implant rehabilitation requires an accurate transfer of the three‐dimensional implant positions to the definitive cast. The precision of this transfer may be influenced by factors such as implant angulation, vertical position, interimplant distance, scanning protocol, ambient lighting, and the technology employed by the intraoral scanner [1, 2]. Achieving a passive fit, in which prosthetic components seat without inducing stress on the implants or surrounding tissues, remains challenging. Cumulative procedural and material‐related errors can compromise prosthetic accuracy, potentially leading to mechanical or biological complications [3].

In clinical practice, anatomical limitations and surgical considerations often necessitate nonparallel implant placement. Angulated implants are frequently used to optimize prosthetic support, although this approach can introduce challenges related to abutment misalignment. The All‐on‐4 concept is a widely adopted strategy in such cases, in which two anterior implants are placed axially, typically in the canine regions, while two posterior implants are tilted distally, generally at 30° or 45°, and positioned anterior to critical anatomical structures such as the mental nerve in the mandible or the maxillary sinus in the maxilla [4]. This configuration maximizes prosthetic support while minimizing the need for extensive grafting procedures. Previous studies have indicated that implant angulation may influence impression accuracy, and comparisons between parallel and nonparallel implant placements have highlighted increased difficulty in achieving a precise prosthetic fit with angulated implants [5, 6].

The accuracy of intraoral scanners is commonly assessed through two key parameters: trueness and precision. Trueness refers to how closely a digital scan represents the actual reference model, while precision indicates the reproducibility of repeated scans under identical scanning conditions. Both parameters are essential for ensuring that digital impressions reliably reflect the true positions of implant analogs in the definitive cast [7].

Although several studies have investigated the accuracy of intraoral scanners for short‐span implant‐supported prostheses, evidence regarding their performance in full‐arch implant‐supported prostheses, particularly at varying vertical implant positions, remains limited [8, 9]. In particular, the effect of crestal versus subcrestal implant positioning on digital impression accuracy has not been fully investigated.

Unlike previous studies that have primarily investigated implant angulation or vertical positioning separately, the present study simultaneously evaluated the effect of the implant vertical position (crestal vs. 2 mm subcrestal) on full‐arch digital impression performance in terms of trueness and precision. In addition, two different intraoral scanning technologies (confocal‐based and structured light systems) were directly compared using interpoint distance analysis against a high‐precision reference scanner, providing a more comprehensive assessment of scanner performance under clinically relevant full‐arch conditions. The present in vitro study was conducted to compare the performance of two intraoral scanners in capturing digital impressions of full‐arch implants positioned at two vertical depths: crestal (0 mm) and 2 mm subcrestal. Accuracy was evaluated in terms of both trueness and precision by analyzing the deviations in implant fixture positions within the virtual casts. The null hypothesis was that neither implant vertical depth nor scanner type would significantly influence trueness or precision and that no interaction would exist between scanner type and implant depth.

## 2. Materials and Method

**Figure 1:**
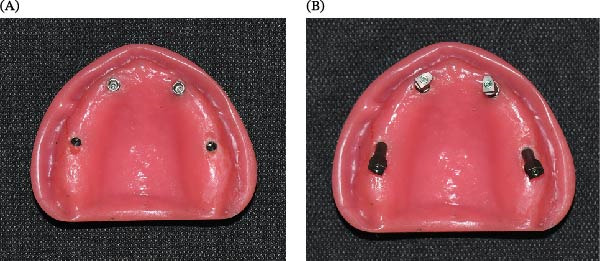
(A) Typodont models showing implant placement: subcrestal and angulated implants at positions 16 and 26, and parallel implants at positions 13 and 23. (B) PEEK scan bodies mounted on the implants for digital scanning.

The present research was conducted experimentally as an in vitro study using two edentulous maxillary typodonts. Holes were created in the models at positions 16, 13, 23, and 26 using a survey milling machine. In one of the typodonts, regular Osstem Implant fixtures (internal connection), Osstem Co., Ltd., South Korea, sized 4 mm, were placed at positions 16 and 26 with a 30° angle and at positions 13 and 23 in a parallel manner. In the other typodont, the Osstem Implant fixtures, sized 4 mm, were placed at positions 16 and 26 with a 30° angle and a 2 mm subcrestal depth and at positions 13 and 23 in a parallel manner at the crestal level (Figure 1A).

Four polyether‐ether‐ketone (PEEK) scan bodies (Osstem Co., Ltd., South Korea; LOT Number: PTE251252) were mounted on the implants using a calibrated torque driver at the manufacturer‐recommended torque of 15 N·cm and positioned for scanning (Figure 1B). Typodonts were scanned using two intraoral scanners: the Planmeca Emerald intraoral scanner (Planmeca Oy, Helsinki, Finland) and the TRIOS 3 intraoral scanner (3Shape, Copenhagen, Denmark), along with a high‐precision laboratory scanner, the 3Shape D810 (3Shape, Copenhagen, Denmark), with a reported precision of 15 μm.

All scans were obtained on edentulous maxillary typodonts under controlled laboratory conditions and not inside an intraoral phantom. During scanning with the Planmeca Emerald, a thin and uniform layer of reflective scanning spray (CEREC Optispray, Dentsply Sirona, Bensheim, Germany) was applied to the typodont surfaces to enhance scan detection. The TRIOS 3 scanner did not require the use of contrast powder.

**Figure 2:**
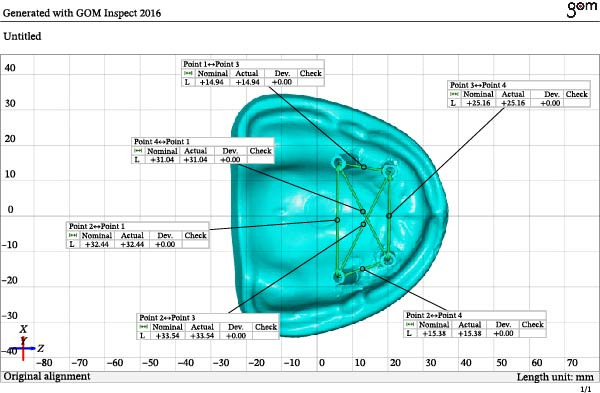
Reference scan in deeply positioned analogs.

An experienced dentist performed all scans in accordance with the manufacturers’ instructions. A standardized arch‐shaped (continuous sweeping) scanning strategy was used for all scans. All scans were performed by a single operator to eliminate interoperator variability. Due to the in vitro design and identical experimental conditions, the scanning order was not randomized. Initially, each typodont was scanned using a 3Shape D810 laboratory scanner to obtain the reference dataset (Figure 2).

Seven scans were obtained for each typodont (14 scans in total per scanner condition). The number of repeated scans was determined based on laboratory feasibility and in agreement with previously published in vitro studies evaluating the intraoral scanner accuracy. However, due to the lack of reliable preliminary data under comparable experimental conditions, a formal a priori sample size calculation was not performed. To complement the statistical interpretation, effect sizes (Cohen’s *d*) were calculated to quantify the magnitude of differences between measurements. To minimize operator fatigue and allow for device cooling, scans were performed at 10‐min intervals.

The proprietary files generated by the 3Shape system were converted into STL format using dedicated software. Subsequently, all STL datasets were imported into GOM Inspect 2016 (GOM GmbH, Braunschweig, Germany) for analysis. For the purposes of this study, the term “model” refers to the STL datasets obtained from the laboratory scanner, which were used solely for the measurement of interpoint distances. No CAD library‐based best‐fit alignment or digital working model creation was performed; therefore, the datasets do not represent digital working models intended for prosthesis fabrication.

A standardized cross‐section was defined 3 mm above the implant platform for all scan bodies to ensure measurement consistency. This cross‐sectional plane was positioned perpendicular to the long axis of each scan body and applied identically across all reference and test datasets within GOM Inspect 2016. At each cross‐section, a best‐fit circle was generated based on the cylindrical contour of the scan body, and the geometric center of this circle was automatically calculated by the software. This point was designated as the central reference point of the corresponding scan body. Consequently, four central points were obtained per dataset, from which six linear interpoint distances were calculated, representing all possible pairwise distances between the scan bodies.

For trueness assessment, the interpoint distances obtained from each TRIOS 3 and Planmeca Emerald scan were compared with those measured in the reference dataset acquired by the 3Shape D810 scanner. All datasets were aligned using the software’s best‐fit algorithm to achieve optimal superimposition. The deviations between each test distance and the corresponding reference distance were calculated, and the mean deviation was defined as the scanner’s trueness.

**Figure 3:**
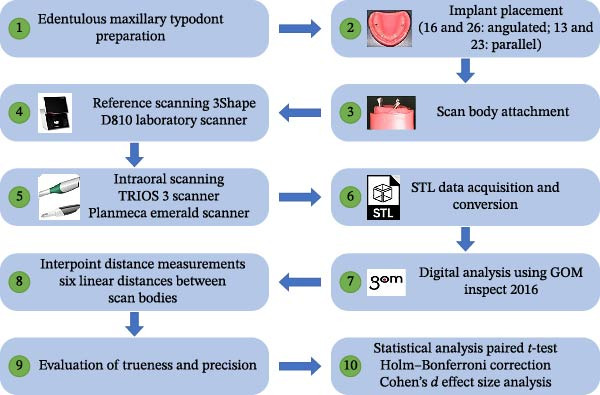
Flowchart of the study procedure.

Precision was evaluated as the standard deviation (SD) of repeated measurements within each group, in accordance with ISO guidelines, where the SD reflects repeatability. Additionally, descriptive statistics including the mean, SD, and standard error were calculated. Normality of the data was assessed using the Kolmogorov–Smirnov test, confirming a normal distribution (*p* > 0.05). A paired *t*‐test was used to compare mean interpoint distance differences between the reference dataset and intraoral scanner measurements for each comparison. To control for type I error due to multiple pairwise comparisons, *p*‐values were adjusted using the Holm–Bonferroni method, and adjusted *p*‐values are reported in the tables. Effect sizes were calculated using Cohen’s *d* for paired samples (Cohen’s *dz*) to quantify the magnitude of differences and are presented in the tables. The level of statistical significance was set at *p* < 0.05. The overall study design and experimental workflow are illustrated in Figure 3.

## 3. Results

All interpoint distances and deviations are reported in millimeters (mm). The Kolmogorov–Smirnov test indicated normal distribution of the data for both intraoral scanners (3Shape and Planmeca Emerald) under subcrestal and crestal implant positions (*p* > 0.05), and therefore parametric analyses were performed. In subcrestal implant models, trueness was assessed by comparing intraoral scanner measurements with the reference laboratory scanner. For the 3Shape scanner, statistically significant differences were observed for distances 1–2, 1–3, and 2–4 after Holm–Bonferroni correction, while the remaining comparisons were not significant. For the Planmeca Emerald scanner, only distance 2–4 remained statistically significant after adjustment, whereas all other comparisons showed no significant differences (Tables 1 and 2).

**Table 1:** Interpoint distance deviations (mm) for the 3Shape scanner in the subcrestal fixture position.

Distances	Mean differences	Standard deviation	*p*‐Value (raw)	*p*‐Value (adjusted)	Cohen’s *d*
2–1	0.07	0.05	0.011	**0.044**	1.40
3–1	−0.04	0.03	0.006	**0.030**	1.33
4–1	0.001	0.05	0.95	1.000	0.02
3–2	0.06	0.06	0.03	0.090	1.00
4–2	0.08	0.02	0.001	**0.006**	4.00
4–3	0.01	0.05	0.67	1.000	0.20

*Note: p*‐Values are adjusted using the Holm–Bonferroni method. Bold adjusted *p*‐Values indicate statistically significant differences after Holm–Bonferroni correction (*p* < 0.05).

**Table 2:** Interpoint distance deviations (mm) for the Planmeca scanner in the subcrestal fixture position.

Distances	Mean differences	Standard deviation	*p*‐Value (raw)	*p*‐Value (adjusted)	Cohen’s *d*
2–1	−0.33	0.63	0.21	1.000	0.52
3–1	0.06	0.12	0.23	1.000	0.50
4–1	0.16	0.33	0.25	1.000	0.48
3–2	0.21	0.46	0.28	1.000	0.46
4–2	0.35	0.07	0.001	**0.006**	5.00
4–3	−0.06	0.34	0.65	1.000	0.18

*Note: p*‐Values are adjusted using the Holm–Bonferroni method. Bold adjusted *p*‐Values indicate statistically significant differences after Holm–Bonferroni correction (*p* < 0.05).

In crestal implant models with 30° angulation, the 3Shape scanner showed significant differences for distances 1–3, 1–4, 2–4, and 3–4 after adjustment, while distance 1–2 was not significant. For the Planmeca scanner, only distances 2–4 remained statistically significant after correction, with all other comparisons showing no significant differences (Tables 3 and 4).

**Table 3:** Interpoint distance deviations (mm) for the 3Shape scanner in the crestal fixture position (30° posterior angulation).

Distances	Mean differences	Standard deviation	*p*‐Value (raw)	*p*‐Value (adjusted)	Cohen’s *d*
2–1	−0.009	0.044	0.63	0.63	0.20
3–1	0.037	0.008	0.001	**0.006**	4.60
4–1	−0.436	0.04	0.001	**0.006**	10.90
3–2	0.107	0.05	0.28	0.56	2.14
4–2	0.266	0.02	0.001	**0.006**	13.30
4–3	−0.113	0.056	0.002	**0.006**	2.01

*Note: p*‐Values are adjusted using the Holm–Bonferroni method. Bold adjusted *p*‐Values indicate statistically significant differences after Holm–Bonferroni correction (*p* < 0.05).

**Table 4:** Interpoint distance deviations (mm) for the Planmeca scanner in the crestal fixture position (30° posterior angulation).

Distances	Mean differences	Standard deviation	*p*‐Value (raw)	*p*‐Value (adjusted)	Cohen’s *d*
2–1	−0.087	0.0445	0.623	0.623	0.19
3–1	0.023	0.066	0.023	0.095	0.35
4–1	0.421	0.348	0.019	0.095	1.21
3–2	0.221	0.356	0.151	0.453	0.62
4–2	0.316	0.193	0.005	**0.030**	1.64
4–3	0.099	0.246	0.33	0.660	0.40

*Note: p*‐Values are adjusted using the Holm–Bonferroni method. Bold adjusted *p*‐Values indicate statistically significant differences after Holm–Bonferroni correction (*p* < 0.05).

Precision was defined as the SD of seven repeated scans for each interpoint distance. In the subcrestal condition, the overall mean SD was 0.043 mm for the ،TRIOS3 scanner and 0.325 mm for the Planmeca Emerald scanner. In the crestal condition, the corresponding values were 0.036 mm for TRIOS 3 and 0.209 mm for Planmeca Emerald. These findings indicate higher repeatability for the TRIOS 3 scanner compared with the Planmeca Emerald scanner in both analog positions.

## 4. Discussion

The present in vitro study evaluated the trueness and precision of two intraoral scanners, TRIOS 3 and Planmeca Emerald, for full‐arch implant scans at different implant angulations and vertical positions. The null hypothesis stated that the scanner type and implant depth would not significantly affect trueness or precision and that no interaction would exist between these variables. Based on the results, the null hypothesis was partially rejected.

Regarding trueness, for subcrestal implants (2 mm depth), the TRIOS 3 scanner showed smaller deviations from the reference scanner (−0.04 to 0.08 mm) compared with the Planmeca Emerald scanner (−0.33 to 0.35 mm), indicating higher trueness of the TRIOS 3 scanner under these conditions. Conversely, for crestal implants, the Planmeca Emerald scanner showed slightly lower mean deviations (−0.087 to 0.421 mm) than the TRIOS 3 scanner (−0.266 to 0.436 mm), suggesting slightly higher trueness at the crestal level.

Analysis of precision, defined as the reproducibility of repeated scans, revealed that the TRIOS 3 scanner consistently exhibited lower SDs than the Planmeca Emerald scanner in both subcrestal and crestal conditions (subcrestal SD: 0.043 vs. 0.325 mm and crestal SD: 0.036 vs. 0.209 mm). These findings indicate greater repeatability of repeated scans with the TRIOS 3 scanner.

Taken together, these findings demonstrate that while trueness varied depending on the implant depth and scanner type, precision was consistently higher for the TRIOS 3 scanner. Therefore, the null hypothesis was partially rejected, highlighting the influence of scanner technology and implant positioning on the full‐arch digital impression performance.

The results are in agreement with previous studies reporting that the scanner performance may be influenced by implant angulation, depth, and scanning protocol. Di Fiore et al. [10] evaluated the accuracy of full‐arch digital impressions using intraoral scanners in implant‐supported prostheses and reported that TRIOS 3Shape and True Definition provided the highest accuracy among the tested systems. Scan‐abut software was used to compare digital impressions with the master model for accuracy assessment. The authors concluded that not all intraoral scanners are suitable for full‐arch implant‐supported prosthetic digital impressions.

Similarly, Amin et al. [11] evaluated full‐arch digital impressions using parallel anterior implants and posterior implants angled between 10° and 15° with the 3M True Definition and CEREC Omnicam scanners. After superimposition of STL datasets with the master cast, they reported higher trueness for digital impressions compared with conventional open‐tray techniques [11]. In the present study, the TRIOS 3 scanner demonstrated better performance for posterior implants with a 30° angulation and 2 mm subcrestal depth. Differences between scanners may be related to the differences in scanning technology. The TRIOS 3 scanner is based on confocal microscopy and ultrafast optical scanning, whereas the Planmeca Emerald scanner uses structured light projection and triangulation. Structured light systems are generally more sensitive to variations in the scanning distance and angulation, which may affect measurement consistency [12].

Ender et al. [13] evaluated the accuracy of full‐arch digital impressions using various scanners and reported that systems such as Lava COS, CEREC Bluecam, and Cadent iTero showed lower accuracy compared with Lava T‐Def, CEREC Omnicam, TRIOS 3Shape, and TRIOS 3Shape Color. In the present study, both TRIOS 3 and Planmeca Emerald demonstrated comparable trueness to the reference scanner across angulated, straight, and subcrestal implant conditions, whereas TRIOS 3 showed higher precision in repeated measurements.

In previous studies, various intraoral scanners have been used, and the type of scanner and the technology employed for the digital impression have been identified as influential factors in the accuracy of the impression [14, 15]. In a study conducted by Vandeweghe et al. [16], 4 intraoral scanners were used to obtain impression data from an edentulous mandibular model, coupled with six implants involving the Lava COS, 3M, CEREC Omnicam, and TRIOS 3Shape scanners. According to recent research findings, the highest accuracy levels were observed in TRIOS and 3M scanners. Additionally, the Lava COS scanner was found to be inappropriate for fabricating implant casts for cross‐arch bridges in edentulous jaws. In the present study, both TRIOS 3 and Planmeca scanners were used. One of these scanners was identical to that used in Vandeweghe et al.’s [16] research. The obtained results demonstrated that the Planmeca scanner showed lower trueness than the 3Shape scanner when the fixtures were simultaneously positioned in angulated and subcrestal conditions (2 mm depth).

The widespread range of errors in implant impression is associated with variations in research methodologies and protocols, types of intraoral scanners and scan bodies, operator’s experience, scanning strategies and methodological changes, implant connection, implant depth, angle, and distance between implants in the study models [17]. In addition, the scanning protocol may have influenced the accuracy of the digital impression. Hence, this protocol should be performed according to the manufacturer’s instructions [16, 18]. In addition to scanner‐related variables, the geometry, material, and design of scan bodies have been reported as significant determinants of digital impression accuracy. Variations in scan body height, diameter, surface characteristics, and material composition (e.g., PEEK versus titanium) may influence scan registration and software alignment, particularly in full‐arch implant cases [19].

In the present study, greater interimplant distances (such as 2–4, 1–4, and 2–3) were generally associated with increased deviations from the reference scanner measurements. However, statistically significant differences were observed only for distances 2–4. These deviations were less pronounced with the TRIOS 3 scanner. The increased scanning span, combined with the absence of fixed anatomical landmarks such as teeth, may have contributed to greater challenges in image‐stitching and reduced trueness over longer distances [20]. Furthermore, errors in distance measurements may be related to limitations in the software‐stitching process and cumulative errors during image data alignment in postprocessing. In this regard, Patzelt et al. [15] reported higher error rates in the distal regions of full‐arch dental scans, attributing this to the accumulation of errors during software processing.

Passive fit refers to the absence of stress transfer to prosthetic components, implants, or the surrounding bone. Achieving passive fit in full‐arch implant‐supported prostheses remains challenging due to cumulative clinical and laboratory errors, which may result in vertical, horizontal, angular, or rotational misfit. Excessive misfit has been associated with mechanical and biological complications, including marginal bone loss and peri‐implant inflammation. Therefore, reducing digital impression errors may contribute to improved fit of full‐arch implant‐supported prostheses [21, 22].

Estimating an appropriate level of fit for implant‐supported restorations remains challenging. Jemt and Lie [23] reported that discrepancies of 100–150 microns may not result in significant clinical complications. Other studies have suggested lower thresholds, ranging from 50 to 75 microns [24, 25]. These values represent cumulative errors from prosthesis fabrication and framework production. It has been reported that the accuracy of full‐arch digital scans is unlikely to be better than ~35 microns [20].

In the present study, TRIOS 3 showed lower mean deviations compared with Planmeca Emerald under the tested implant conditions. Papaspyridakos et al. [26] reported a mean error of 29 μm for TRIOS when scanning 15° angulated implants, which was lower than the values observed in the present study. This difference may be attributed to variations in implant angulation, number of implants, implant design, and scan body characteristics.

Most in vitro studies have reported linear and 3D deviations exceeding 100 microns [10, 27, 28]. Some studies have reported lower values under controlled conditions [11, 29]. Differences between studies may be related to methodological variations and differences in evaluation criteria used to assess digital impression accuracy.

The present study contributes to the existing literature by providing a direct comparison of two intraoral scanning technologies under the combined conditions of full‐arch implant angulation and vertical positioning. Compared with previous reports focusing on isolated variables, this study evaluates the combined influence of the implant position and scanner technology on both trueness and precision, which may provide a more comprehensive assessment of digital impression accuracy.

## 5. Limitations

This study was conducted under in vitro conditions, which do not fully replicate the complex intraoral environment. Factors such as saliva, soft tissue resilience, intraoral humidity, patient movement, temperature fluctuations, and limited intraoral access were not simulated. These variables may influence the intraoral scanning performance, particularly in full‐arch implant impressions, and may introduce additional sources of error under clinical conditions. Therefore, caution should be exercised when extrapolating the present findings to in vivo settings, and future clinical studies are recommended to validate these results under real intraoral conditions [30].

Another limitation of this study is the use of linear interpoint distance measurements instead of three‐dimensional deviation analysis. Although linear measurements are widely used in in vitro studies on full‐arch implant scanning accuracy and allow clinically interpretable comparisons between predefined reference points, they do not fully capture spatial (three‐dimensional) deviations. Therefore, future studies incorporating 3D deviation analysis are recommended to provide a more comprehensive assessment of scanning accuracy.

Finally, the relatively small number of repeated scans may have reduced the statistical power to detect small differences between groups. Accordingly, findings related to minor deviations should be interpreted with caution.

## 6. Conclusion

Based on the research limitations, the results were as follows:

1. The TRIOS 3 scanner demonstrated higher trueness and precision for subcrestal and angulated implants.
2. The Planmeca Emerald scanner exhibited slightly higher trueness for crestal implants.
3. Both scanners demonstrated small deviations in trueness compared with the reference scanner in angulated, subcrestal, and parallel full‐arch implant conditions.
4. Precision was consistently higher for the TRIOS 3 scanner across most implant positions.

## Author Contributions

**Sina Safari**: conceptualization, investigation, formal analysis, supervision. **Neshat Atashpareh**: conceptualization, methodology, investigation, writing – original draft preparation. **Hamed Bahrami Maleki**: methodology, data curation, writing – review and editing.

## Funding

No funding was received for this research.

## Disclosure

All generated content was carefully reviewed, revised, and approved by the authors, who take full responsibility for the final version of the manuscript. No author is affiliated with any funding organization through employment, consultancy, or any other financial relationships.

## Ethics Statement

This study was an in vitro investigation and did not involve human participants or animals; therefore, ethical approval was not required.

## Conflicts of Interest

The authors declare no conflicts of interest.

## Data Availability

The data that support the findings of this study are available from the corresponding author upon reasonable request.
